# Drug resistant tuberculosis in Saudi Arabia: an analysis of surveillance data 2014–2015

**DOI:** 10.1186/s13756-018-0306-4

**Published:** 2018-01-22

**Authors:** Maha Al Ammari, Abdulrahman Al Turaiki, Mohammed Al Essa, Abdulhameed M. Kashkary, Sara A. Eltigani, Anwar E. Ahmed

**Affiliations:** 1 King Abdullah International Medical Research Center (KAIMRC)/King Abdulaziz Medical City(KAMC), Ministry of National Guard - Health Affairs, Riyadh, Saudi Arabia; 2 College of Pharmacy, King Saud bin Abdulaziz University for Health Sciences, Riyadh, Saudi Arabia; 3 Ministry of Health Kingdom of Saudi Arabia, Riyadh, Saudi Arabia; 4 College of Public Health and Health Informatics, King Saud bin Abdulaziz University for Health Sciences, Riyadh, Saudi Arabia

**Keywords:** Mdr-Tb, RR-Tb, Tuberculosis, Anti-TB drugs, Saudi Arabia

## Abstract

**Background:**

There is limited data that investigates the national rates of drug-resistant tuberculosis (TB) in Saudi Arabia.

This study aimed to estimate the rates of multi-drug-resistant tuberculosis (MDR-TB), rifampicin-resistant tuberculosis (RR-TB), and monoresistance (MR) in Saudi Arabia.

**Methods:**

A retrospective cohort study was conducted on all TB cases reported to the National TB Control and Prevention Program (NTCPP) registry at the Saudi Ministry of Health between January 1, 2014 and December 31, 2015. A total of 2098 TB patients with positive TB cultures were included in the study. Subgroup analyses and multivariate binary logistic regression models were performed with IBM SPSS 23.0.

**Results:**

Of the total TB cases, 4.4% (95% CI: 3.59%–5.40%) were found to have MDR-TB. The rates of MR were 3.8% (95% CI: 2.99%–4.67%) for ethambutol, 5.4% (95% CI: 4.50%–6.49%) for pyrazinamide, 10.2% (95% CI: 5.89%–11.52%) for isoniazid, 11% (95% CI: 9.70%–12.43%) for streptomycin, and 5.9% (95% CI: 4.90%–6.96%) for rifampicin. The high rates of MDR and RR-TB were found among the younger age group, female gender, and those who had a previous history of TB. We also discovered that renal failure tends to increase the risk of rifampicin resistance.

**Conclusions:**

National TB data in Saudi Arabia shows that the rate of MDR-TB was similar to the global rate reported by the World Health Organization (WHO). It is a relatively high rate as compared to Western countries. The proportion of MDR/RR-TB patients tends to be higher in the younger age group, female gender, and in patients with a previous history of TB treatment. Effective strategies for prevention of all multi-drug-resistant TB cases are warranted.

## Background

Tuberculosis (TB) is a major public health concern in Saudi Arabia’s health system [1]. Despite the implementation of prevention and control measures, TB remains a public health concern in several of the developed world’s health systems [2–5]. The World Health Organization (WHO) reported around 10 million newly diagnosed TB cases in 2016 [6].

According to WHO, TB has been ranked Number 11 of the top leading causes of death in Saudi Arabia. A total of 64,345 new TB cases were reported in Saudi Arabia during a period of 20 years (1991 to 2010) [7]. Although TB can be treated in most cases, patient and health-system challenges exist regarding proper utilization of TB treatment, compliance [8], and direct observation of each TB patient under treatment [9]. Unfortunately, TB bacilli may develop resistance to Anti-TB drugs. [10].

MDR-TB (MDR-TB) is defined as resistance to isoniazid and rifampicin, the two most potent anti-TB drugs [11]. Developing resistance to anti-tuberculosis medications has immense implications on the management of TB by 1) increasing the treatment duration, 2) having to use second-line medications with broader side-effects profiles, 3) the increased cost of therapy, and 4) the lower success rates (82% for drug-susceptible TB, 52% for MDR-TB, 28% for XDR-TB) [6, 12, 13].

Globally, of all TB patients diagnosed in 2015, 4.6% of the new TB cases were found to have MDR-TB, in addition to 21% of the previously treated patients, and it was estimated that there were 480,000 new cases of MDR-TB worldwide [6]. The Saudi region-specific rates of MDR-TB were reported between 1 and 5% [14–16]. A single national study reported an overall MDR-TB rate of 4% [17]. To date, there has been no other national study that investigates the MDR-TB rate in Saudi Arabia. This epidemiological study aimed to estimate the rates of MDR-TB, RR-TB, and monoresistance to anti-TB drugs in Saudi Arabia. We also assessed the association between the demographic and clinical characteristics and the rate of high MDR-TB and RR-TB in the Saudi population.

## Methods

A retrospective cohort study was conducted on all TB cases reported to the National TB Control and Prevention Program (NTCPP) registry at the Saudi Ministry of Health between January 1, 2014 and December 31, 2015. The NTCPP registry is a data registry at the Saudi Ministry of Health, where all suspected and confirmed TB cases are registered from all Saudi Arabian regions, with all related variables that enable researchers and stakeholders to retrieve and analyze any data at any period of time. The authors used a retrospective design because it can be useful in identifying the factors associated with the high rates of drug-resistant TB using large-scale existing data. We assessed the drug-resistant TB over a two-year period (January 1, 2014 through December 31, 2015). The Institutional Review Board (IRB) approval was obtained from King Abdullah International Medical Research Center (KAIMRC) in December 2016. All registered TB cases in the NTCPP registry from January 2014 through December 31, 2015 were reviewed. The eligibility criterion was defined as TB cases with positive TB cultures as culture is considered the gold standard method that provides viable organisms to perform DST. DST is the definite diagnostic tool for confirming resistance of *Mycobacterium tuberculosis* against isoniazid, rifampicin, ethambutol, pyrazinamide, and streptomycin as per program guidelines. No subsample was selected as all TB patients fulfilling the eligibility criteria were included in the study. We extracted demographic data (age, gender, nationality, and place of residence); co-morbidity (renal disease, HIV, diabetes, lung disease, and immunosuppression); type of TB (pulmonary or extra pulmonary); history of previous TB treatment; and the drug susceptibility test for isoniazid, rifampicin, ethambutol, pyrazinamide, and streptomycin.

### Statistical analysis

We analyzed the data using IBM SPSS Statistics for Windows, version 23 (IBM Corp., Armonk, N.Y., USA). Summary statistics were used to describe the sample characteristics (Table 1). The rates of multi-drug-resistant tuberculosis (MDR-TB), rifampicin-resistant tuberculosis (RR-TB), and monoresistance (MR) were described by percentage and 95% confidence intervals (CI). We estimated the rates of MDR-TB and RR-TB by each of the variables.

**Table 1:** Sample characteristics *N* = 2098

Characteristics	*Levels*	Mean	SD
Age/year		36.6	15.9
		n	%
Gender	*Male*	1449	69.1
	*Female*	649	30.9
Occupation	*Driver*	147	7
	*Housemaid*	246	11.7
	*Housewife*	172	8.2
	*Handcraft*	48	2.3
	*Student*	148	7.1
	*Unemployed*	283	13.5
	*Prisoner*	123	5.9
	*Other*	931	44.4
Nationality	*Saudi*	879	41.9
	*Non-Saudi*	1219	58.1
Region	*Center*	557	26.5
	*West*	796	37.9
	*East*	340	16.2
	*South*	290	13.8
	*North*	115	5.5
TB site	*Pulmonary*	1901	90.6
	*EPTB*	197	9.4
Diabetes	*Yes*	267	12.7
	*No*	1831	87.3
HIV	*Yes*	45	2.1
	*No*	2053	97.9
Lung diseases	*Yes*	54	2.6
	*No*	2044	97.4
Chronic renal failure	*Yes*	19	0.9
	*No*	2079	99.1
Immunosuppressive	*Yes*	13	0.6
	*No*	2085	99.4
Previous TB treatment	*Yes*	143	6.8
	*No*	1955	93.2

*SD* Standard deviation, *HIV* human immunodeficiency virus, *TB* tuberculosis

#### Subgroup analyses

Chi-square/Fisher’s Exact were used to test the associations between the sample characteristics across MDR-TB and RIF-resistance (Table 2).

**Table 2:** Bivariate factors associated with MDR-TB and RR-TB

		MDR-TB 93(4.4%)						RR-TB 123(5.9%)					
						95% CI for OR						95% CI for OR	
Characteristics	*Levels*	Mean Difference	SE Difference	P	OR	Lower	Upper	Mean Difference	SE Difference	P	OR	Lower	Upper
Age/year		3.8	1.4	0.006^a^	.983	.968	.998	2.547	1.5	.084	.989	.977	1.001
						95% CI for OR						95% CI for OR	
		n	%	P	OR	Lower	Upper	n	%	P	OR	Lower	Upper
Gender	*Male*	60	4.1	0.332	1.240	.803	1.916	84	5.8	0.848	1.039	.702	1.537
	*Female*	33	5.1					39	6		1		
Occupation	*Driver*	8	5.4	0.984	1.133	.523	2.455	10	6.8	0.918	1.060	.530	2.119
	*Housemaid*	10	4.1		.834	.414	1.680	12	4.9		.744	.394	1.407
	*Housewife*	6	3.5		.712	.299	1.695	7	4.1		.616	.277	1.371
	*Handcraft*	2	4.2		.856	.201	3.639	3	6.3		.968	.292	3.205
	*Student*	6	4.1		.832	.349	1.986	7	4.7		.721	.323	1.608
	*Unemployed*	11	3.9		.796	.406	1.561	17	6		.928	.532	1.617
	*Prisoner*	5	4.1		.834	.325	2.144	7	5.7		.876	.391	1.962
	*Other*	45	4.8		1			60	6.4		1		
Nationality	*Saudi*	34	3.9	0.286	1.264	.821	1.946	51	5.8	0.920	1.019	.704	1.475
	*Non-Saudi*	59	4.8		1			72	5.9		1		
Region	*West*	43	5.4	0.014^a^	.937	.585	1.500	54	6.8	0.004^a^	.892	.587	1.356
	*East*	5	1.5		.245	.094	.635	6	1.8		.220	.093	.524
	*South*	9	3.1		.525	.247	1.116	17	5.9		.764	.427	1.367
	*North*	4	3.5		.591	.205	1.705	4	3.5		.442	.155	1.258
	*Center*	32	5.7		1			42	7.5		1		
TB site	*Pulmonary*	87	4.6	0.320	1.527	.659	3.539	113	5.9	0.622	1.182	.608	2.296
	*EPTB*	6	3		1			10	5.1		1		
Diabetes	*Yes*	9	3.4	0.367	1.378	.685	2.775	14	5.2	0.645	1.144	.646	2.027
	*No*	84	4.6		1			109	6		1		
HIV	*Yes*	92	4.5	1.000	2.064	.281	15.148	121	5.9	0.720	1.347	.322	5.625
	*No*	1	2.2		1			2	4.4		1		
Lung diseases	*Yes*	2	3.7	1.000	.825	.198	3.442	4	7.4	0.555	1.294	.460	3.644
	*No*	91	4.5		1			119	5.8		1		
Chronic renal failure	*Yes*	2	10.5	0.205	2.570	.585	11.292	4	21.1	0.022^a^	4.392	1.435	13.439
	*No*	91	4.4		1			119	5.7		1		
Immunosuppressive	*Yes*	1	7.7	0.446	1.805	.232	14.032	2	15.4	0.175	2.951	.647	13.463
	*No*	92	4.4		1			121	5.8		1		
Previous TB treatment	*Yes*	36	25.2	0.001^a^	11.203	7.069	17.755	41	28.7	0.001^a^	9.181	6.005	14.038
	*No*	57	2.9		1			82	4.2		1		

^a^Significant at α = 0.05; *OR* odds ratio, *TB* Tuberculosis, *HIV* human immunodeficiency virus, *MDR-TB* multi-drug-resistant tuberculosis, *RR-TB* rifampicin-resistant tuberculosis, *EPTB* extra-pulmonary tuberculosis, *SE* (standard error)

#### Multivariate analyses

We used multivariate logistic models to identify the factors related to MDR-TB and RIF-resistance (Table 3). All variables assessed by the subgroup analyses were included in the multivariate models. The strength of the relation was assessed using unadjusted and adjusted odds ratios OR and aOR)and 95% CI (Table 2 and Table 3, respectively). In all analyses, the significance level was determined at *P* ≤ 0.05.

**Table 3:** Multivariate factors associated with MDR-TB and RR-TB

		MDR-TB				RR-TB			
				95% CI for aOR				95% CI for aOR	
Factor		P	aOR	Lower	Upper	P	aOR	Lower	Upper
Age		0.032^a^	0.98	0.962	0.998	0.047^a^	0.98	0.970	1.000
Female	*Male*	0.015^a^	2.21	1.170	4.181	0.044^a^	1.78	1.015	3.120
Occupation: Driver	*Other*	0.232	1.68	0.718	3.932	0.285	1.51	0.708	3.237
Occupation: Housemaid	*Other*	0.116	0.49	0.200	1.193	0.147	0.55	0.246	1.234
Occupation: Housewife	*Other*	0.036^a^	0.33	0.114	0.932	0.010^a^	0.28	0.109	0.744
Occupation: Hand Craft	*Other*	0.390	1.93	0.430	8.654	0.241	2.11	0.606	7.342
Occupation: Student	*Other*	0.113	0.44	0.157	1.217	0.037^a^	0.37	0.144	0.940
Occupation:Unemployed	*Other*	0.395	0.72	0.338	1.534	0.267	0.70	0.370	1.317
Occupation: Prisoner	*Other*	0.771	0.86	0.313	2.369	0.584	0.79	0.330	1.868
Saudi	*Non-Saudi*	0.921	0.97	0.569	1.664	0.583	1.14	0.716	1.813
West	*Center*	0.624	0.88	0.522	1.477	0.371	0.81	0.514	1.282
East	*Center*	0.038^a^	0.35	0.131	0.942	0.005^a^	0.28	0.115	0.685
South	*Center*	0.181	0.57	0.254	1.296	0.552	0.82	0.437	1.556
North	*Center*	0.710	0.81	0.274	2.418	0.273	0.55	0.189	1.601
TB Type: Pulmonary	*EPTB*	0.480	1.38	0.565	3.375	0.766	1.11	0.548	2.260
Diabetes	*No*	0.835	0.92	0.411	2.052	0.874	0.95	0.486	1.847
HIV	*No*	0.469	2.34	0.234	23.392	0.416	2.14	0.343	13.353
Lung diseases	*No*	0.549	0.63	0.138	2.868	0.789	0.85	0.271	2.693
Chronic renal failure	*No*	0.102	4.01	0.759	21.231	0.004^a^	6.61	1.860	23.495
Immunosuppressive	*No*	0.738	1.56	0.116	21.008	0.262	3.18	0.420	24.113
Previous TB treatment	*No*	0.001^a^	12.08	7.325	19.927	0.001^a^	9.33	5.920	14.717
(Intercept)		0.003	0.02			0.002	0.04		

^a^Significant at α = 0.05; *aOR* adjusted odds ratio, *TB* Tuberculosis, *HIV* human immunodeficiency virus, *MDR-TB* multi-drug-resistant tuberculosis, *RR-TB* rifampicin-resistant tuberculosis, *EPTB* extra-pulmonary tuberculosis. The percentages of correct classification were 95.6% for MDR-TB and 94.1% for RR-TB

## Results

A total of 6753 patients included in NTCPP registry from January 1, 2014 through December 31, 2015 were reviewed. Around 4655 patients were excluded due to non-availability of culture results, or non-availability of rifampicin-susceptibility test results. A total of 2098 patients with positive TB cultures enrolled in the study for the final analysis.

The mean age was 36.6 (SD = 15.9) years. Of the TB cases, 69.1% were male, 11.7% were housemaids, and 41.9% were Saudi. About 38% of these TB cases occurred in the Western region of Saudi Arabia. Pulmonary tuberculosis was common in TB patients (90.6%), while 9.4% had extra-pulmonary tuberculosis (EPTB). More details can be found in Table 1. The rate for MDR-TB in TB patients studied was 4.4% (95% CI: 3.59%–5.40%), while the resistance rate was 3.8% (95% CI: 2.99%–4.67%) for ethambutol, 5.4% (95% CI: 4.50%–6.49%) for pyrazinamide, 5.9% (95% CI: 4.90%–6.96%) for rifampicin, 10.2% (95% CI: 5.89%–11.52%) for isoniazid, and 11% (95% CI: 9.70%–12.43%) for streptomycin.

According to subgroup analyses (Table 2), the resistance rates for MDR/RR-TB were low in the Eastern region. The resistance rates for MDR-TB and RR-TB were high in patients with previous anti-TB treatment, and chronic renal failure was associated with a higher rate of RR-TB. There was no association regarding gender, age, TB site, nationality, or HIV status. According to multivariate logistic models (Table 3), the female gender is more likely to have MDR-TB and RR-TB (2.21 and 1.78 times) as compared to the male gender, respectively. As age increases by 1 year, MDR-TB and RIF-resistance tend to decrease by 2%.

In comparison to patients with no previous anti-TB treatment, MDR-TB and RR-TB were 12.08 and 9.33, respectively, times more likely to occur in patients with previous TB treatment. Patients from the Eastern region are less likely to develop MDR-TB and RR-TB by 65% and 72% respectively, compared with patients from the Central region. Compared to patients with no renal failure, patients with renal failure were 6.61 times more likely to have RR-TB. Housewives, compared to other occupations, were 67% and 72% less likely to have MDR-TB and RR-TB, respectively.

## Discussion

Our data showed that the rate of MDR-TB in Saudi Arabia is within the global average as per the 2016 WHO report [6]. Similar to a previous Al-Hajoj et al. study, our study revealed that the Western and Central regions showed the highest rates of MDR-TB and Eastern region was the lowest [17]. The high rates in the Western region could be due to the presence of the two biggest holy sites (Mecca and Medina), which are visited by millions of Muslim from all around the world, including from countries where TB is highly endemic. During Hajj season, TB is the most frequent cause of hospitalization [18]. In the Central region we expected the higher rate could be due to the presence of four tertiary care hospitals in addition to the MOH central hospital and the chest hospital, that serve as referral centers from the periphery for all suspected and confirmed TB cases.

Comparing our result with the Al-Hajhoj study, there were slightly higher rates of MDR-TB (4.4% vs. 4.0%) and RR-TB in our study (5.8% vs. 5.3%), respectively. Comparing the rate of MDR-TB in the newly treated patients, the Al-Hajhoj study showed 1.8% of newly treated patients had MDR-TB, while our study showed a higher rate of MDR-TB (2.9%) among the newly diagnosed patients [17]. Our study showed consistent results with several published studies regarding previously treated patients and the risk of MDR-TB [17, 19–24].

Our study also revealed that each one-year increase was associated with a 2% decrease in MDR-TB. This was similar to what was seen in a European meta-analysis, which showed that patients younger than 65 years of age were associated with a higher risk of MDR-TB [19] and in an Ethiopian study, which showed that patients younger than 25 years of age had an increased risk of MDR-TB [20]. This association could be explained by the fact that younger patients have lower compliance to the medication compared with the elderly.

In our study, the female gender was associated with a higher rate of MDR-TB compared with males. This finding contradicted the results of the previously mentioned meta-analysis, which showed that males have a higher chance of developing MDR-TB when compared to females [19].

This study has several limitations: the data were collected retrospectively, and due to the nature of the study, we did not include modifiable factors such as patient compliance and appropriateness of regimen. These factors could be important in developing anti-TB resistance as is shown in a study conducted in Turkey in 2004 [24]. Data were not available for close contact with other MDR-TB patients, alcohol use, and monthly income in our patients, which may be important factors for a high rate of MDR-TB as was shown in the Mulu study [20]. No data on intravenous drug abuse was collected, however it was found to be associated with MDR-TB in a 2014 study conducted in Portugal [23]. Despite these limitations, the study determined the overall MDR-TB rate and TB patients with high risk in Saudi Arabia. The study may be helpful to policymakers wanting to address the rising concern of MDR-TB in Saudi Arabia.

## Conclusion

National TB data in Saudi Arabia shows the rate of MDR-TB is in accordance with global rates, however, it is a relatively high rate when compared to Western countries. The proportion of MDR/RR-TB cases tends to increase in younger age group, female gender, and in patients with previous TB treatment. The rates of MDR/RR-TB varied between regions in Saudi Arabia, with the Eastern region reporting the lowest MDR-TB rate. Further studies are required to understand the association between the suggested high risk and drug-resistant TB. It will help in addressing the early identification of the drug-resistant TB patients and their management.
